# The effect of group A streptococcal carrier on the epidemic model of acute rheumatic fever

**DOI:** 10.1186/s12976-019-0110-8

**Published:** 2019-09-10

**Authors:** Natsuda Yokchoo, Nichaphat Patanarapeelert, Klot Patanarapeelert

**Affiliations:** 1 Department of Mathematics, Faculty of Applied Science, King Mongkut’s University of Technology North Bangkok, Bangkok, 10800 Thailand; 2 Department of Mathematics, Faculty of Science, Silpakorn University, Nakorn Pathom Province, 73000 Thailand

**Keywords:** Acute rheumatic fever, Group A streptococcus, Mathematical model, Carriers

## Abstract

**Background:**

Group A streptococcus (GAS) is the most frequent cause of bacterial pharyngitis in school-aged children. The postinfection sequel as acute rheumatic fever (ARF) and rheumatic heart disease that cause morbidity and mortality among young people is public health concerns in several developing countries. Asymptomatic carriage state of GAS is not fully understood in terms of host and bacterial factors. Although the ability of transmitting GAS of the asymptomatic carriers is relatively low, they may present the reservoir of the epidemic. A fraction of GAS carriers is difficult to estimate in practice and may greatly vary between populations. Understanding the role of carriage on the transmission dynamic of GAS is important for assessing the public health impact of the ARF.

**Method:**

This study investigates the effect of GAS carriers on both the transmission and dynamic of ARF cases by using a mathematical model.

**Result:**

We derive the sufficient conditions for which the GAS can spread or extinct from the naive population under the variation of the fraction of symptomatic cases over the incidence of GAS. The threshold is possible to occur in general, but the last condition which is rather restrictive and involves parameter uncertainty. The increasing of carriers in the endemic state leads to the reduction in magnitude of the reproduction number and the number of ARF patients. We demonstrate that the adjustment of parameters can be carried out by the use of endemic state and some specific data.

**Conclusion:**

We show theoretically that the presence of asymptomatic carriers may induce the epidemic threshold and reduce the virulence of GAS and the prevalence of ARF.

## Background

Acute Rheumatic Fever (ARF) is an inflammatory disease which developed as a sequel of group A *β*-hemolytic streptococcal (GAS) pharyngitis [1–3]. The illness by ARF is ranging from fever, the inflammation of joints, carditis to the chorea. The possible consequence as chronic rheumatic heart disease is considerable as life-threatening, leading to heart failure and death. Among the ARF patients, the rheumatic heart disease is a major cause of morbidity and mortality among children and young people (between 5-15 years of age). Nowadays, ARF may be considerable as a rare disease in some regions, it is significant public health concerns around the world, especially in developing countries [4, 5]. The incidence of ARF varies greatly between 0.08–20 per 100,000 during the beginning of the 20th century [2]. Also, the predicted trend shows the significant variation between counties. Prevention and the treatment of ARF directly involve the effective diagnosis and antibiotic treatment of GAS [3]. Untreated streptococcal infections or experiencing ARF patients receive an increasing risk for ARF development and its recurrence.

Streptococcus pyogenes is responsible for 10 to 30% of pharyngitis in children [6, 7]. It was estimated that 5–15% of school-aged children harbor GAS in pharynx but do not develop symptom. These may be characterized as a carriage state of GAS [8–10]. Although the exact definition of carrier remains elusive [11, 12], the most common feature involving in the transmission is asymptomatic. The identification of the carrier can be carried out only by a bacterial culture or a rapid antigen detection test [13, 14]. Since the carriers do not have GAS symptom, except for the common symptoms caused by viral infection, the evaluation of the fraction of carriers among the population under study may be obscure.

GAS can be transmitted mainly via respiratory droplets. Patients who are asymptomatic carriers are thought to have a low ability to transmit the infection to the others [13, 15]. Since the carriers have no respiratory symptoms such as cough or coryza for the majority of the time that they are carriers, the possibility of spread of GAS to the environment is relatively low. Little increased possibility may caused by the respiratory symptoms that developed by virus infection [11]. Autoimmune response to GAS infection in genetically predisposed individuals is believed to be the cause of ARF development in GAS patients [1]. The risk is increased for untreated GAS patients and the patient who had ARF. On the other hand, the risk of ARF development in carriers is not confirmed, due to the sparsity of direct evidence. Although carriage is thought not to be a risk for ARF, switching GAS carriage *emm* types may be at risk of ARF [13].

This study aims at contributing to the hypothesis that the presence of carriers can reduce the virulence of GAS during the epidemic in the general population. Moreover, if the ARF cases is assumed to be directly proportional to the GAS patients, the reduction in ARF prevalence caused by the presence of carriers could be as a byproduct or secondary impact. However, the conclusion may not be straightforward since the carriers normally constitute an important reservoir of GAS infection [11, 16]. To understand the role of carrier on the epidemic of GAS and ARF, we develop a mathematical model for the transmission dynamic of GAS incorporating with the ARF compartment. According to the determinism, the key measures as the basic reproduction number and the endemic state are emphasized. The first quantity is used to address whether the threshold property driven by carrier arises, and the latter will be used to explain the role of parameter variation and provide the framework for the estimation of parameters when dealing with the data.

## Method

### Mathematical model

We use the standard compartmental SIS model to describe the infection of GAS and the development of ARF. According to [12], the symptomatic infections (or infectious class) is labeled by *I*, and asymptomatic carriers (or carrier) is labeled by *C*. Although the carriage state can be defined in other different ways (see [11]), it is reasonable to use the asymptomatic state as a gauge for discriminating the infected compartment in order to prevent the vagueness. Since the carriers cannot be cured from the treatment with an appropriate antimicrobial agent, we will not consider the recovery of carriers for the present model.

We assume that a fraction, *α*, of GAS infections result in illness followed by recovery with no immunity, and on the other hands, the fraction, 1−*α*, will lead to persistence of GAS in the carrier state. Since the transmission by carriers is relatively low, the force of infection by carrier is always lower than another. Although carriers harbor GAS for long periods, they may be at risk to develop symptomatic infection later. Hence, the flow rate to infectious class is possible. The effectiveness of treatment for the symptomatic case may not be perfect, it is possible that a little portion of incomplete eradication can be accounted for carrier. Therefore, even with the very low rate, we assumed that the interchange between infectious and carrier groups is possible.

The infectious group and carriers are at risk to develop ARF with different conditions and degree. The carrier is known to have a little risk to develop ARF, while the infectious individual has a greater chance. For simplicity, the risk to develop ARF is homogeneous among infectious individuals and carriers. In our model, a person who has ARF can be treated and goes into the susceptible state. As in [15], we furnish the model by letting *A* be the state of ARF. In summary, the model equations are given by

1) (2) (3) (4 $$\begin{array}{@{}rcl@{}} \frac{dS}{dt}&=&\Lambda +\gamma_{2} A+\theta\gamma_{1} I-\left(\beta_{1} I+\beta_{2}C+\!\mu\right)S,  \\ \frac{dI}{dt}&=& \alpha S\left(\beta_{1} I+\beta_{2}C\right)+\epsilon C\,-\,(\gamma_{1}+\delta_{1}+\mu)I, \\ \frac{dC}{dt}\!&=&\!(1\,-\,\alpha) S\left(\beta_{1} I\,+\,\beta_{2}C\right)\,+\,(1\!\,-\,\theta)\gamma_{1} I\,-\,(\epsilon\,+\,\delta_{2}\,+\,\!\mu)C, \\ \frac{dA}{dt}&=&\delta_{1} I+\delta_{2} C-(\gamma_{2}+\mu)A. \end{array} $$

Here, all variables describe the number of individuals in each disease state at time *t*, and the definitions of model parameters are described in Table 1. We note that the transmission coefficients *β*_*i*_,*i*=1,2 are defined as the product of the contact rate *ϕ* and the transmission probabilities per single contact *p*_*i*_, divided by the total population size. It is easy to see that all variables of the system (1)-(4) are non-negative for all *t*>0. Let *N*=*S*+*I*+*C*+*A* be a total population. By adding all equations together, we obtain

$$\frac{dN}{dt} =\Lambda-\mu N. $$

**Table 1:** Definition of model parameters and the range

Symbol	Definition	Range	Reference
*γ*_1_	Recovery rate conditioned on perfect treatment	0.1-1 day ^−1^	[10, 15, 17]
*γ*_2_	Recovery rate of an ARF patient	0.1-1 day ^−1^	[1]
*θ*	Efficacy of GAS treatment	0.8-1	[6, 15]
*ϕ*	Contact rate	1-10 times*day −1	[12]
*p*_1_	Transmission probability by a symptomatic case	0.89-0.99	[12]
*p*_2_	Transmission probability by a carrier	0.001-0.05	[12]
*ε*	Transfer rate of carrier to infectious state	variable	[12, 13]
*α*	A symptomatic fraction over the new infections	variable	-
*δ*_1_	ARF development rate from GAS infectious sate	0.0027-0.08 day ^−1^	[2, 15]
*δ*_2_	ARF development rate from GAS carrier state	variable	[11]

Thus, the population tends to a steady-state at *Λ*/*μ*, as *t*→*∞*. Throughout, we will focus the dynamic of the system (1)-(4) only on the steady-state population.

## Results

### The basic reproduction number of GAS epidemic

The basic reproduction number *R*_0_ is defined as the average number of secondary infections by an index case introduced into the whole susceptible population during the infectious period [18]. The quantity is important in epidemiology, which depends on the method of derivation. For the deterministic model, *R*_0_ exhibits the threshold of the epidemic, i.e., if *R*_0_<1, then the disease eventually dies out, and if *R*_0_>1, then the epidemic occurs. Since *R*_0_ is a function of model parameters, the threshold can be viewed as driven by a critical parameter.

We first derive *R*_0_ of the GAS epidemic by using the next generation method [19, 20]. After setting the right hand sides of Eqs. (1)-(4) equal to zero, we obtain the disease-free equilibrium:

5 $$\begin{array}{@{}rcl@{}} \left(S^{0},I^{0},C^{0},A^{0}\right)=\left(\frac{\Lambda}{\mu},0,0,0\right). \end{array} $$

At the disease-free equilibrium, we define the matrices

$$\begin{array}{*{20}l} F=&\left[ \begin{array}{cc} \alpha S^{0}\beta_{1} & \alpha S^{0}\beta_{2} \\ (1-\alpha)S^{0}\beta_{1} & (1-\alpha)S^{0}\beta_{2} \end{array} \right],\\ V=&\left[ \begin{array}{cc} (\gamma_{1}+\delta_{1}+\mu) & -\epsilon \\ -(1-\theta)\gamma_{1} & \epsilon+\delta_{2}+\mu \end{array} \right]\end{array} $$

which describe the rate of appearance of new infections and the rate of other transitions, respectively. Hence, the next generation matrix can be calculated as *FV*^−1^, and we can obtain *R*_0_ from its spectral radius as

6 $$ {}R_{0}=R_{1}\left(\frac{\alpha(\delta_{2}+\mu)+\epsilon} {\delta_{2}+\mu+\kappa\epsilon} \right) + R_{2}\left(\frac{(1-\kappa\alpha)(\delta_{2}+\mu)} {\delta_{2}+\mu+\kappa\epsilon}\right)  $$

where

7 $$\begin{array}{@{}rcl@{}} R_{1} &=& \frac{\beta_{1} S^{0}}{\delta_{1}+\mu+\gamma_{1}},  \\ R_{2} &=& \frac{\beta_{2} S^{0}}{\delta_{2}+\mu}, \\ \kappa &=& \frac{\delta_{1}+\mu+\theta\gamma_{1}}{\delta_{1}+\mu+\gamma_{1}}.  \end{array} $$

Just as in [16], the form of *R*_0_ is the sum of two terms, each of which describes the transmitability of the group weight by a term describing the fraction of symptomatic infection and other transitions. In our case, *R*_1_ represents the basic reproduction number of GAS when there is no carriers, and *R*_2_ is the basic reproduction number in the absence of infectious individuals.The parameter *κ* lies in (0,1], which is a linear function of the effectiveness of treatment *θ*.

### Threshold analysis of GAS epidemic

The existence of carriers induces the silent epidemic for GAS. However, it is not clear to what extent the carrier size has an impact. More precisely, we may ask if the variation in an incidence of carriers can bring the epidemic threshold? and if so, on what conditions? In this section, we will address such questions by analyzing the threshold property of *R*_0_. The goal is to determine the condition at which the threshold induced by the fraction *α* exists.

We first assume that *R*_1_>*R*_2_. This is reasonable assumption at least in the broad sense, namely the relative rate of flow outs from the infectious state does not high enough to overcome the relative transmission rate:

8 $$ \frac{\beta_{1}}{\beta_{2}} > \frac{\delta_{1}+\mu+\gamma_{1}}{\delta_{2}+\mu}.  $$

We note that, albeit the above relation is reversed, the following results can be obtained in the same manner.

From (6), *R*_0_ can be viewed as a linear function of *α* as

9 $$  R_{0}(\alpha) = \xi \alpha + R_{0}(0),  $$

where the slope and the interception are given by

$$\begin{array}{@{}rcl@{}} \xi &=& \frac{(\delta_{2}+\mu)}{(\delta_{2}+\mu+\epsilon\kappa)}(R_{1}-\kappa R_{2}),  \\ R_{0}(0) &=& \frac{\epsilon R_{1}+(\delta_{2}+\mu)R_{2}}{\delta_{2}+\mu+\kappa \epsilon}.  \end{array} $$

Since *κ*<1, it is clear that *R*_1_−*κR*_2_>0. Under the present assumption, *R*_0_ is linearly increasing with *α*. Since 0≤*α*≤1, it follows that

$$R_{0}(0)\le R_{0}(\alpha) \le R_{0}(1). $$

Hence, the epidemic threshold induced by *α* can occur only subject to the conditions,

$$R_{0}(0) < 1\ \ \text{and}\ \ R_{0}(1) > 1. $$

To examine through parameter conditions such that the above relations are satisfied, we first observe that

$$\begin{array}{@{}rcl@{}} R_{0}(1) &=& \frac{R_{1}(\delta_{2}+\mu+\epsilon)+R_{2}(1-\kappa)(\delta_{2}+\mu)} {\delta_{2}+\mu+\kappa\epsilon},  \\ &<& \frac{R_{1}}{\kappa}\left(\frac{\kappa(\delta_{2}+\mu+\epsilon)+(1-\kappa)(\delta_{2}+\mu)} {\delta_{2}+\mu+\kappa\epsilon} \right) = \frac{R_{1}}{\kappa}.  \end{array} $$

So, we must provide that *R*_1_>*κ*, otherwise the threshold would never exist. On the other hands, by (9), it can be seen that *R*_0_(0)>*R*_2_. Thus, we must put *R*_2_<1, so that the threshold is possible.

Consider

$$\begin{array}{@{}rcl@{}} 1-R_{0}(0) &=& \frac{(\delta_{2}+\mu)(1-R_{2})-\epsilon(R_{1}-\kappa)}{\delta_{2}+\mu+\kappa\epsilon}  \\ &<& \frac{(\delta_{2}+\mu)(1-R_{2})}{\delta_{2}+\mu+\kappa\epsilon}  \\ &<& \frac{(\delta_{2}+\mu)(R_{1}-\kappa R_{2})}{\delta_{2}+\mu+\kappa\epsilon} = \xi.  \end{array} $$

By Eq. (9), we just now proved that *R*_0_(1)>1 without further condition. To complete the required sufficient conditions, we only force

10 $$ \epsilon(R_{1}-\kappa) < (\delta_{2}+\mu)(1-R_{2}).  $$

This implies that, *R*_0_(0)<1.

In summary, the parameter conditions such that the threshold of GAS epidemic induced by the parameter *α* exists are given by

- *R*_2_<1,
- *R*_1_>1, and
- *ε*(*R*_1_−*κ*)<(*δ*_2_+*μ*)(1−*R*_2_).

The last condition seems to be more restrictive than the first two conditions since the force of infection by the symptomatic patient is normally stronger than by the asymptomatic carrier. To satisfy the last condition, it may be sufficient to assume that the rate of progression to symptomatic infection of carriers is sufficiently low.

Suppose that the above conditions are satisfied. The critical value of *α*, that is *α*_*c*_ can be determined by solving an equation *R*_0_(*α*_*c*_)=1. Here, we have

11 $$ \alpha_{c} = \frac{(\delta_{2}+\mu)(1-R_{2})-\epsilon(R_{1}-\kappa)}{(\delta_{2}+\mu)(R_{1}-\kappa R_{2})}.  $$

Therefore, *R*_0_<1, if *α*<*α*_*c*_, and *R*_0_>1 if *α*>*α*_*c*_. Theoretically speaking, to contain the spread of GAS, the infections per unit time must produce at least 1−*α*_*c*_, a fraction of carriers.

### Parameter values and the threshold of epidemic

To demonstrate the threshold by model solutions, a set of parameter values must be derived. Nevertheless, the relevant information about the parameter estimation is sparse. Some parameters may be difficult to estimate, for example, the fraction of new infections leading to the carrier, 1−*α*, and the transfer rate from carriers to ARF group, *δ*_2_. Here, we sought to use the most informative data available in the literature to determine the ranges of parameter values (see Table 1 for the range and references). We choose a particular value for each parameter from its possible range, so that the existence conditions for the threshold are satisfied. The limitations and the reasons for using the baseline values are described as follows.

Throughout, the population size is set to be one thousand with an average life span of 70 years. Thus, the recruitment rate *Λ* is 0.0391 per day. As in previous work [12], the transmission rates, *β*_*i*_,*i*=1,2, are defined as the product of contact rate and the transmission probability per single contact divided by the total population:

$$\beta_{i} = \frac{\phi p_{i}}{N}. $$

The contact rate, *ϕ* is given by two times per day, while the transmission probabilities are assumed to be 0.9 and 0.001, when a contact made by an infectious individual (*p*_1_) and made by a carrier (*p*_2_), respectively.

The treatment of GAS infection is usually a 10-day course of oral penicillin or a single dose of intramuscular benzathine benzylpenicillin [1]. An active antibiotic treatment results in negative throat cultures within 24 h in more than 80% of patients [6]. By this we infer that the efficacy of GAS treatment is high that is between 80% and 100%. We choose the midpoint of the treatment efficacy, that is 90%. The recovery rate conditioned on the perfect treatment is approximated as the inverse of the treatment duration. We assume that it is between one to 10 days. Since the treatment is assumed to be perfect, we choose *γ*_1_=0.7.

The outflow rate from the carrier to infectious state, *ε* is not exactly known. The presence of the subsequent episodes of symptomatic pharyngitis may vary in the degree of virulence and the factor determining whether the individual becomes a GAS carrier [21]. The lack of information about the duration of carriers makes the estimation difficult. The interval of long period (one to four years) was used in modeling work [12], while the interval of short period (3–34 weeks) is evident in empirical study [13]. The latter means the period at which the children carried a single *emm* type. To fulfill the existence condition (iii), the value must be sufficiently low. We assume that its average is one year, thus *ε*=1/365, per day.

Although, the pathogenesis of ARF remains incompletely understood, the immune response to GAS infection is believed to play a prominent role [21]. The evidence shows that the patients who had ARF had significant rises in the antibody to the extracellular antigens [11], which increases the risk to the recurrent ARF. On the other hand, if a GAS infection is not properly treated, ARF may develop after two to three weeks [15]. This is supported by the observation of 92% of individuals who developed ARF within a month of acquiring GAS (see reference in [13]). If *δ*_1_ is modeled by the inverse of such period, then its value is between 0.04 to 0.07 per day. Since the rate is per capita, it should be multiplied by the risk factor which lies between 0.3−3*%* [15]. However, the baseline value is assumed to be 0.05, to ease the threshold condition. The development rate of ARF among carriers is not known in the literature. We hypothesize that the carrier has a relatively low risk for ARF, namely *δ*_2_<*δ*_1_. In this case, we choose *δ*_2_=0.02.

The recovery rate of ARF, *γ*_2_ is approximated as the inverse of an average treatment duration. The treatment of ARF has three primary goals, and the duration depends on the severity of clinical symptom [1]. Treatment of GAS infection is the first priority, usually followed by the variety of anti-inflammatory medications. The medications are given until the inflammatory markers normalized, usually within 4 to 6 weeks [4]. Since the model assumed that an individual who recovered from ARF becomes susceptible to the GAS infection, the duration of GAS treatment is quite preferable to the choice of the recovery rate of ARF for the present model.

**Fig. 1:**
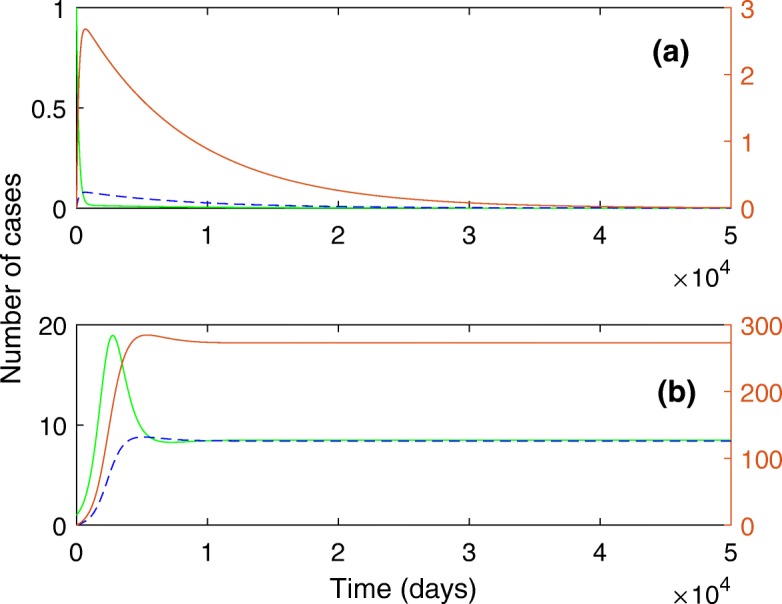
Below and above the epidemic threshold. The solution curves for *I*(*t*) (green line), *C*(*t*) (red line) and *A*(*t*) (dash line). **a** when *α*<*α*_*c*_. **b** when *α*>*α*_*c*_. The parameter values are described in the text

According to such parameter values (see Table 2), we calculate *R*_1_=2.4,*R*_2_=0.09 and *κ*=0.9. By these results all conditions for threshold are satisfied so that we get *α*_*c*_=0.3. Based on theoretical prediction if we put *α*=0.1, then the disease must die out (See Fig. 1a), and if we put *α*=0.5, then the disease persists (see Fig. 1b). Throughout the numerical calculation, we use the initial condition, *S*(0)=999,*I*(0)=1,*C*(0)=0, and *A*(0)=0.

**Table 2:** Sensitivity index of *R*_0_ and *α*_*c*_

Parameter	Baseline value	Sensitivity index of *R*_0_	Sensitivity index of *α*_*C*_
*p*_1_	0.9	+0.9355	−1.5106
*p*_2_	0.001	+0.0645	−0.1042
*ϕ*	2	+1	−1.6148
*α*	0.9146	+0.6193	−
*θ*	0.9	−0.1247	+0.2013
*μ*	0.3914×10^−4^	−0.5303×10^−3^	+0.8564×10^−3^
*ε*	0.0027	+0.1816	−0.2933
*δ*_1_	0.05	−0.0633	+0.1022
*δ*_2_	0.02	−0.2457	+0.3967
*γ*_1_	0.7	−0.8721	+1.4083

### Sensitivity analysis

We perform sensitivity analysis of *R*_0_ and *α*_*c*_ in order to determine the influence of the model parameters and to inform the degree of uncertainty on a particular set of parameter values. Here, the local sensitivity indices are calculated for both outputs at a common set of baseline parameter values. Suppose that *p* is an input parameter. The normalized forward sensitivity index of the output, *u* is calculated by

12 $$ \frac{p}{u}\frac{\partial u}{\partial p}.  $$

In Table 2, 10 from 12 input parameters are tested for the sensitivity of *R*_0_, and 9 parameters are tested for the sensitivity of *α*_*c*_. We note that *R*_0_ does not depend on *Λ*, and *γ*_2_, and, the sensitivity index of *R*_0_ to *α* is calculated at *α*_*c*_. Thus, the index determines how the value of *R*_0_ changes from unity. For both outputs, we find that the contact rate, *ϕ* is the most sensitive parameter. The other important parameters are *p*_1_,*γ*_1_, and *α*, respectively. Except for *γ*_1_, such three parameters have a positive influence to *R*_0_, and the direction is reversed for *α*_*c*_. The result can be interpreted as follows. Since *R*_0_=1, at *α*=*α*_*c*_, the sensitivity index for *α*_*c*_ can be calculated as

13 $$ \frac{p}{\alpha_{c}}\frac{\partial \alpha_{c}}{\partial p} = -\frac{p}{\alpha_{c}} \frac{\partial R_{0}/\partial p}{\partial R_{0}/\partial \alpha}.  $$

From Eq. (9), it is easy to verify that the partial derivative of *R*_0_ with respect to *α* is positive. Thus, the sign of sensitivity index of *α*_*c*_ is opposite to the sign of sensitivity index of *R*_0_. If an increase of one parameter leads to increasing of *R*_0_, the value of *α*_*c*_ is decreased. The latter means that the area for *R*_0_>1 is extended.

### GAS persistence and the implication of ARF

#### The existence of endemic state

Persistence of a disease pathogen in a population, including demographic effects is determined by the so-called endemic state, the positive equilibrium point of the model. Here, we will show that the existence of this equilibrium depends only on the parameter *R*_0_, that is *R*_0_>1.

Let (*S*^∗^,*C*^∗^,*I*^∗^,*A*^∗^) be an endemic state of the system (1)-(4). By solving a system of algebraic equations directly, we obtain

$$S^{*} = \frac{S^{0}}{R_{0}}. $$

From Eq. (2), we can write

$$C^{*} = \left(\frac{\gamma_1+\delta_1+\mu}{\alpha \beta_{2} S^{*}+\epsilon}\right)\left(1-\frac{\alpha R_{1}}{R_{0}}\right)I^{*} $$

By rearranging the expression of *R*_0_, we have

14 $$ R_{0} = \alpha R_{1} + \left(\frac{1-\alpha \kappa}{\delta_{2}+\mu+\kappa \epsilon} \right)\left(\epsilon R_{1} + R_{2}(\delta_{2}+\mu)\right).  $$

This shows that *R*_0_>*αR*_1_. Thus, if *I*^∗^ is positive, then so is *C*^∗^.

From Eq.(4), we obtain

15 $$  A^{*} = \frac{\delta_{1} I^{*}+\delta_{2} C^{*}}{\gamma_{2}+\mu}.  $$

Substituting this into *S*^0^=*S*^∗^+*I*^∗^+*C*^∗^+*A*^∗^, we have

16 $$  S^{0}\left(1-\frac{1}{R_{0}}\right) = I^{*}+C^{*}+\frac{\delta_{1} I^{*}+\delta_{2} C^{*}}{\gamma_{2}+\mu}.  $$

We have seen that the endemic state can be uniquely determined only if the positive value of *I*^∗^, exists. From the above equation, if *R*_0_>1, then *I*^∗^ can be solved for positive value, followed by the rest of the variables. Thus, we have shown that the endemic state exists and unique.

#### The effect of GAS carriers on ARF

So far, we have seen that the carriers when incorporated into the system could reduce the total virulence of GAS epidemic comparing to which the infections completely produce the infectious cases under the same parameters condition. We now suppose that *R*_0_>1, which guarantees the persistence of GAS followed by ARF. Analyzing the role of GAS carriers on the endemic state of ARF is nontrivial. Instead of using the explicit expression, we first focus on the impact of two relevant parameters *θ* and *α*, the treatment efficacy and the probability that a new infection resulting in the infectious state. Since the remaining fractions contribute to the growth rate of the carrier group, increase of such parameter values is, in turn reduce the growth rate. In an extreme case, i.e., *θ*=1 and *α*=1, the carrier is absolutely absent from the system. In this case, our model recovers the model of [15]. Thus, the impact on ARF is comparable when such couple parameters are continually decreased.

**Fig. 2:**
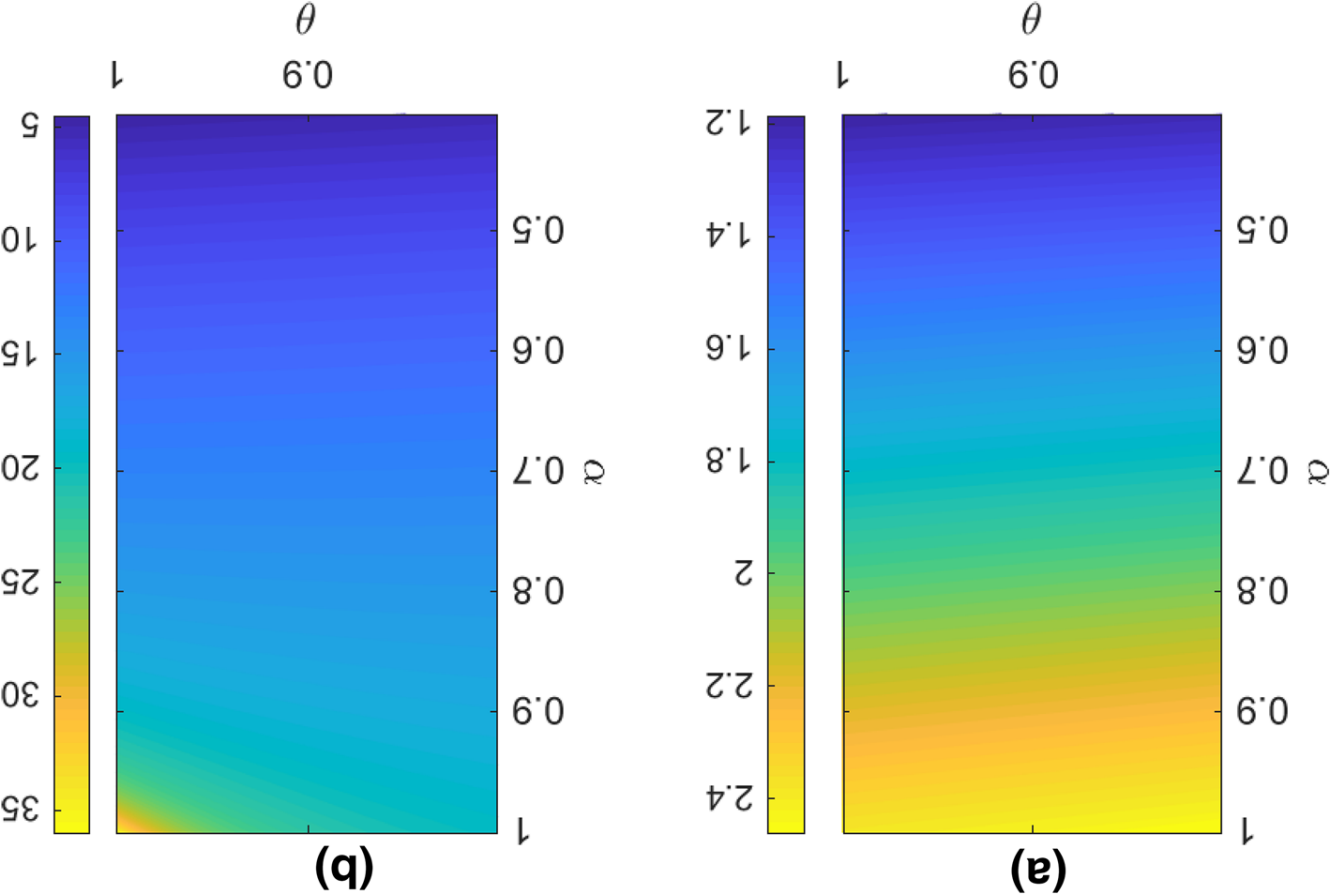
The effect of carriers on the endemic state of ARF. Subject to the GAS outbreak condition, the left panel shows the variation of *R*_0_, and the right panel shows the endemic state of ARF. The top right conner is a point associated with the absence of carriers. **a** R_0_. **b** A^∗^

Based on the set of parameter values used in the previous case, we focus on the variations of *θ* and *α* in a small region that gives *R*_0_>1 (see Fig. 2a). Within that parameter space the endemic state of ARF is depicted in Fig. 2b. It is seen that in the absence of carrier, the model predicts highest level of ARF. As the growth rate of carriers increases, the number of ARF patients becomes decreasing. The reason behind this is that we have set that *δ*_2_<*δ*_1_. The development rate to ARF is assumed to be proportional to the infected population, and that of carrier group is relatively low.

#### Model calibration

The numerical results under a trial of parameters seems against the general perception, especially the ARF cases are looking overestimated (see Fig. 1). To be more realistic, but specific, we roughly adjust a set of parameter values according to data obtained by a field study and a basic information about ARF. A simple procedure is developed based on the use of endemic state.

The rate of carriage depends highly on how carriers are defined and population under study. Among school surveys, it was found that the number of carriers is between 8% to 40%. Here, we refer particularly to a 4-year longitudinal study of school-aged children [13]. From laboratory analysis of throat cultures of samples, the mean prevalence of carriers among general population is about 16%. By connecting this with the model, we deduce that *C*^∗^≈0.16*S*^∗^.

It is also recognized that 0.3*%* to 3% of people will develop ARF following a GAS infection [1]. To be simplified, we use the average value, that is about 1.65*%* of infectious individuals. Hence, we deduce that *A*^∗^≈0.0165*I*^∗^.

By using these two approximations, it might be able to solve for two unknown parameters, say *α* and *δ*_2_. Due to the nonlinear relationship between parameters, the analytic formula of solution is difficult to obtain. Moreover, the solution of nonlinear system does not always exist. By the existence we mean that the solution must be nonnegative real and less than or equal to one. To deal with this difficulty, we solve the equations numerically. It can be seen that the solution does not exist by using the baseline of parameter values in Table 2. Thus, reconsidering the choice of baseline parameters is required.

After numerical trails, values of *γ*_1_,*p*_1_,*ε* and *δ*_1_ are adjusted as follows (see Table 3). The recovery rate of GAS patient has increased from 0.7 to 0.9 per day. The transmission probability per contact from the symptomatic GAS patient is reduced from 0.9 to 0.5, which is below the range. The transfer rate from the carrier to infectious class is increased by reducing the time that an individual spent in a carrier state before changing to symptomatic infection from one year to a month. We note that this period satisfies the short duration given by [13]. The last parameter that was changed is the rate at which ARF develops in the infectious class. As mentioned earlier, we multiplied the baseline value by 0.1 which represents the probability that a GAS patient develops ARF. This is because GAS remains present in the throat even after adequate treatment in about 10% of cases [2].

**Table 3:** Sensitivity of parameter esitmation

Parameter	Adjusted Baseline	Sensitivity index of *δ*_2_	Sensitivity index of *α*
*γ*_1_	0.9	−16.9270	−2.0013
*θ*	0.9	−16.9270	−3.0889
*p*_1_	0.5	−17.2278	−2.1795
*p*_2_	0.001	+0.0901	+0.00943
*ϕ*	2	−17.3327	−2.1955
*ε*	0.0333	−0.2771×10^−4^	−0.1046
*γ*_2_	0.7	+1.5110	−0.4006×10^−1^
*δ*_1_	0.005	−0.7633	+0.0061
*Λ*	0.0391	−0.2924×10^−2^	−0.4385×10^−3^
*μ*	0.3914×10^−4^	−0.2924×10^−2^	−0.4385×10^−3^

By using the adjusted baseline, we find *α*=0.9971, and *δ*_2_=0.0025. This implies that, to match the endemic state of the model with the real data, only about 0.3*%* of GAS incidence are the carriers, and the rate at which those carriers develop ARF is low, i.e., 0.0025 per day. It is observed that the predicted value of *δ*_2_ is less than of *δ*_1_. Moreover, the new set of parameter values does not fulfill the third existence condition of the threshold, which implies that *R*_0_>1, for all *α*. As a result, we obtain that *R*_1_=1.105,*R*_2_=0.7731, and *R*_0_=1.2233.

Clearly, the result of estimation has a degree of uncertainty. Once the baseline values are changed, the estimation gives the new couple *α*, and *δ*_2_. One may ask how sensitive is the estimation to the baseline parameters, and which parameters influence. We perform the sensitivity analysis for the parameter estimation based on perturbation of fixed point estimations [22]. A series of tests are performed on the adjusted baseline in Table 3. Since it is impossible to determine the exact formulas of *α*, and *δ*_2_ in terms of other parameters, the sensitivity index of *α* with respect to the parameter *p* is approximated as

17 $$ \frac{p}{\alpha} \frac{\partial \alpha}{\partial p} \approx \frac{p}{\alpha} \frac{\Delta \alpha}{\Delta p} = \left(\frac{\Delta p}{p} \right)^{-1} \left(\frac{\alpha(p+\Delta p)-\alpha}{\alpha} \right).  $$

For 10 input parameters to be tested, changing is taken as 1% in positive direction except for *ϕ*, and *p*_1_, the change is backward in 1%. This is because the solution of the nonlinear system does not exist when the forward change is taken on such parameters. The result shows that the four highest sensitive parameters are *ϕ*,*p*_1_,*θ*, and *γ*_1_. The magnitude of sensitivity index of *δ*_2_ is relatively high, since the value of *δ*_2_ is relatively small. The sign of index indicates that the estimated values of *α*, and *δ*_2_ decrease as the transmission rate of infectious class is decreased. On the other hand, their values decrease as the recovery rate of GAS increases.

## Conclusions and discussion

Threshold analysis was exemplified for studying the role of carrier on GAS epidemic. The fraction of new infections that develop clinical symptoms, *α* is assumed as a key measure to determine the outbreak of GAS under the certain conditions. Specifically, if *R*_1_>*R*_2_, then *R*_0_ increases with *α*. It should be noted that this is not necessary in general. Suppose that *κR*_2_>*R*_1_, we find that *R*_0_ decreases with *α*. In this case, it is easy to verify that the analytic results can be obtained in a similar way under the reverse direction.

In addition to *α*, the effect of carrier might be expressed through other ways. The parameters *κ* and *ε*, for instance, involve in the transfer rates of carrier class. The first one, however, slightly vary close to unity due to the fact that the treatment of GAS in general is almost perfect. Unlike the second parameter, the value of *ε* can be much vary across the different populations. For example, in a school-aged survey over a 4 years period, the duration of carrier state ranged from 10 weeks to 127 weeks. The more prolong for the period of carrier, the more reducing the outflow rate. In the very small value of *ε*, it tends to satisfy the third condition for the threshold to be existing. On the other hand, if the value of *ε* is not small as estimated in the last section, the further analysis of epidemic threshold incorporating with the first two conditions may be required.

In summary, we have shown that the presence of carrier theoretically reduces the virulence of GAS and the incidence of ARF as the secondary impact. Due to the lack of data, the analysis of dynamic properties of endemic state is limited. Nevertheless, the formulas for endemic state was shown to be sufficiently useful in capturing the epidemiological aspect of the carrier state in a specific population. However, the more systematic procedure of parameter estimation is required to better explain the existing data. Moreover, the model modification and extension are also noted for future development. As far as the pathogenesis of ARF is ongoing research, the gap of new modeling approach in this topic is still held. The stochastic model of ARF development should be focused, and also one can account for the infection profiles of individual for both GAS and ARF, which usually increases the risk of ARF recurrence. In this case, the heterogeneity of the population should be paying attention on both the transmission of GAS and the development of ARF.

## Data Availability

Not applicable.

## Abbreviations

ARF: Acute rheumatic fever
GAS: Group A streptococcus
